# Is the initiation of selfing linked to a hermaphrodite’s female or male reproductive function?

**DOI:** 10.1007/s00265-020-2816-3

**Published:** 2020-03-17

**Authors:** Philipp Kaufmann, Lukas Schärer

**Affiliations:** 1 Department of Environmental Sciences, Zoological Institute, University of Basel, Vesalgasse 1, 4051 Basel, Switzerland; 2 Present Address: Department of Evolutionary Biology, Evolutionary Biology Centre, Uppsala University, Norbyvägen 18D, 752 36 Uppsala, Sweden

**Keywords:** Initiation of selfing, Hermaphrodite, Reproductive strategy, *Macrostomum hystrix*, Selfing vs. outcrossing, Mating behaviour

## Abstract

**Abstract:**

There is an ongoing debate about whether simultaneous hermaphrodites capable of selfing should prefer selfing over outcrossing or vice versa. While many theoretical models predict a transmission advantage for alleles that favour selfing, empirical studies often reveal low selfing rates. Despite these considerations, the underlying mechanisms that determine reproductive strategies in simultaneously hermaphroditic animals are poorly understood. In our study on the facultatively selfing free-living flatworm, *Macrostomum hystrix*, we ask whether the initiation of selfing, as inferred from the differential spatial distribution of received sperm, is linked to an individual’s female or male reproductive function. Specifically, the initiation of selfing could (i) be linked to the male function, when an individual is unable to donate sperm to others and hence donates sperm to self, or it could (ii) be linked to the female function, when an individual fails to receive sperm from others—and hence is unable to fertilize its eggs via outcrossing—thus inducing it to self-fertilize. We experimentally created a social environment that allowed focals to outcross via sperm donation, but simultaneously prevented them from receiving sperm—by pairing them with a partner lacking the male copulatory organ—so that fertilization of the focal’s eggs was restricted to selfing. Our results suggest that such focals generally do not initiate selfing, while we readily observe selfing in isolated worms. This suggests that in isolated *M. hystrix*, it is the male function that is linked to the initiation of selfing, likely due to a lack of opportunities for sperm donation.

**Significance statement:**

A variety of simultaneously hermaphroditic animals are capable of reproducing via both selfing and outcrossing. While the reproductive choices of such animals can be modelled by the joint action of genetic (e.g. inbreeding depression) and ecological factors (e.g. partner availability), experimental evaluation of theoretical results is often lacking. By manipulating the social environment of focal individuals, we here provide evidence that explores the respective role that the co-occurring male and female sex functions have on the initiation of selfing in a simultaneously hermaphroditic flatworm species. Specifically, our results suggest that the initiation of selfing is linked to the worm’s male function. Insights about which function is linked to the initiation of selfing may ultimately help to better understand reproductive decisions in simultaneous hermaphrodites.

**Electronic supplementary material:**

The online version of this article (10.1007/s00265-020-2816-3) contains supplementary material, which is available to authorized users.

## Introduction

Hermaphroditism is the dominant sexual system in flowering plants (Renner and Ricklefs 1995), but also very widespread in animals (Ghiselin 1969; Charnov 1979; Jarne and Auld 2006). An organism is considered hermaphroditic when it is capable of producing both female and male gametes during its lifetime, and if the production of both gamete types occurs at the same time, then the organism is considered a simultaneous hermaphrodite. The co-occurrence of both gamete types may open up the possibility for selfing, which is the fusion of a female and a male gamete of a single genetic entity (colony or individual) (Jarne and Charlesworth 1993). However, not all simultaneously hermaphroditic species self-fertilize (Fischer 1980; Barrett 1988; Jarne and Charlesworth 1993; Jarne and Auld 2006) and even within species that are capable of selfing, differences in selfing rates have been reported between populations (e.g. Ward et al. 2005; Tedder et al. 2015). While some species (or populations) do reproduce via selfing, others do so by outcrossing, while yet others have mixed systems that combine both reproductive strategies (Jarne and Charlesworth 1993; Goodwillie et al. 2005; Jarne and Auld 2006). This raises the question about whether a hermaphrodite that is capable of selfing should preferentially do so or whether it should rather prefer outcrossing. Darwin (1876) observed many morphological traits of simultaneously hermaphroditic plants, whose functions were suggested to only support outcrossing, leading him to conclude that outcrossing is preferred in simultaneously hermaphroditic plants, a conclusion supported by observing offspring of higher quality resulting from outcrossing (i.e. growing bigger in size and producing more seeds). Although Darwin acknowledged that selfing might be a useful reproductive assurance strategy, he questioned the value of selfing compared to outcrossing whenever the latter strategy was available.

Using a mathematical model, Fisher (1941) showed a large transmission advantage of an allele that induces selfing. Based on Fisher’s model, such an allele would outcompete an allele for outcrossing and therefore spread in an initially outcrossing population, since a selfing individual is both mother and father to its offspring. Fisher’s calculations were based on plants, assuming non-assortative mating and that all genotypes can contribute equally to the pollen pool for outcrossing. Later, the spread of a selfing allele was also supported under more realistic conditions, in models considering pollen discounting and lower quality of inbred individuals (Nagylaki 1976; Holsinger et al. 1984; Wells 1979). In species that are capable of selfing, but usually have high outcrossing rates, this therefore demands for strong selective forces that counteract the transmission advantage of selfing. For example, for outcrossing populations in a changing environment, recombination permits faster adaptation (Schemske and Lande 1985), while such advantages of genetic recombination are more limited in highly inbred populations (Charlesworth and Wright 2001). However, spatial and temporal variations in the environment alone are currently not considered to be sufficient to overcome the transmission advantage of a selfing allele (Maynard Smith 1978; Jarne and Charlesworth 1993).

Instead, it was concluded that inbreeding depression in self-fertilized offspring probably constitutes the major force opposing selection for increased selfing rates (Charlesworth and Charlesworth 1979; Jarne and Charlesworth 1993; Husband and Schemske 1996). Inbreeding depression mainly results from two genetic mechanisms (reviewed in Charlesworth and Charlesworth 1987; Charlesworth and Willis 2009), namely overdominance (a loss of heterozygosity at overdominant loci) and partial dominance (inbred individuals become increasingly fixed for recessive and partially recessive deleterious alleles). However, when inbreeding occurs frequently, the effect of the latter mechanism may eventually become small, as deleterious recessive alleles are selected against and eventually drop out of the population (genetic purging) (reviewed in Crnokrak and Barrett 2002; but see also Byers and Waller 1999). Models considering the genetic costs (inbreeding depression) and benefits (transmission advantage) of selfing predict disruptive selection against intermediate selfing rates, favouring the evolution of either fully outcrossing or fully selfing populations (Lande and Schemske 1985). However, other models that take ecological factors, such as reproductive assurance in to account, also allow for intermediate selfing rates to be a stable strategy (Stebbins 1957; Massol and Cheptou 2011).

Why selfing rates differ among and within species has now been discussed for many years and still remains a question that draws a lot of attention (Goodwillie et al. 2005; Jarne and Auld 2006; Barrett 2013; Wright et al. 2013). Here, we tackle this topic from a different angle. Rather than asking why a hermaphroditic animal is outcrossing or selfing, we try to understand whether the initiation of selfing is linked to either the male or the female function of a hermaphroditic individual, thereby exploring aspects of the underlying processes that lead to selfing.

**Fig. 1:**
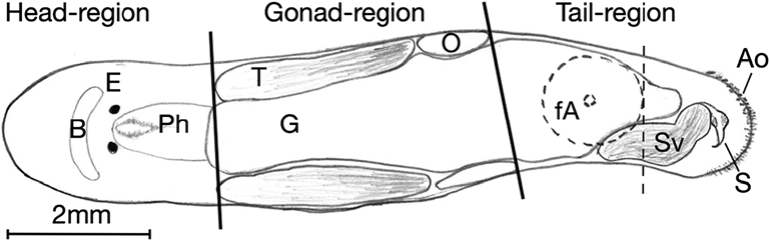
Schematic anatomy illustration of *Macrostomum hystrix*. The solid lines divide the worm into three body regions, namely the head, gonad and tail region. The dashed line indicates the intended cutting level. Also indicated are some of the organs that are visible in a transparent adult *M. hystrix*: The brain (B), eyes (E), pharynx (Ph), testes (T), ovaries (O), gut (G), female antrum (fA), seminal vesicle (Sv), stylet (S) and adhesive organs (Ao)

In trying to answer this question, we used the free-living flatworm *Macrostomum hystrix*, a highly suitable model organism to study selfing in simultaneously hermaphroditic animals. *M. hystrix* mates via hypodermal insemination, penetrating the epidermis of a mating partner with a needle-like sclerotized male copulatory organ (the stylet) and injects sperm into the partner’s parenchyma (the tissue between epidermis and gut) (Schärer et al. 2011). When raised in groups, *M. hystrix* appears to predominately reproduce via outcrossing, but it is also capable of selfing if there are no mating partners available (Ramm et al. 2012). Specifically, selfing likely occurs by hypodermic self-insemination into the anterior body regions (Ramm et al. 2015), as inferred from differential spatial distribution of parenchymal sperm between isolated and grouped worms (see also next paragraph). Once the sperm are injected, they then presumably migrate through the parenchyma towards the site of fertilization, likely in the ovary region of the flatworm (Fig. 1). In many species, the selfing rate probably depends on access to partners (and their gametes) to outcross with (Tsitrone et al. 2003; Kalisz et al. 2004). However, inbred progeny of *M. hystrix* may show inbreeding depression, in that selfing worms produce fewer hatched offspring per time unit and their offspring tend to die more frequently compared to the outcrossed parental generation (Ramm et al. 2012). Moreover, as predicted by theoretical models for populations that encounter inbreeding depression by selfing (Tsitrone et al. 2003), isolated *M. hystrix* have been shown to delay the onset of selfing by several days compared to individuals raised in groups (Ramm et al. 2012), a phenomenon called delayed selfing (Escobar et al. 2011).

The body of *M. hystrix* is highly transparent and therefore allows direct observation and scoring of received sperm in living worms under the light microscope (note that such scoring only refers to transferred sperm in the parenchyma, i.e. sperm injected by a partner or self- insemination, further called ‘parenchymal’ sperm; it this does not include any own sperm that is stored in the testes or the seminal vesicle before transfer). Different spatial distribution patterns for received sperm in worms kept in isolation (i.e. presumably selfing) and worms maintained in triplets (i.e. presumably outcrossing) have been observed in *M. hystrix* (Ramm et al. 2015). In particular, isolated individuals had high sperm scores in the head region (Fig. 1) and decreasing values towards the tail region, while individuals in triplets revealed the reverse pattern, with low sperm scores in the head region and high scores in the tail region (Ramm et al. 2015). This led Ramm et al. (2015) to conclude that self-insemination takes place mainly into the head region, while sperm received from a partner is injected mainly into the tail region. These differences in the spatial distribution patterns of parenchymal sperm can therefore serve as a proxy for distinguishing between parenchymal autosperm (self-inseminated sperm) and parenchymal allosperm (sperm received from a partner) and thus between selfing and outcrossing in *M. hystrix*.

A hermaphrodite has two possible routes to fitness (Schärer et al. 2014), namely via the male function by donating sperm to fertilize available eggs (including its own) and via the female function by receiving sperm (including its own), allowing it to fertilize its own eggs. While in *M. hystrix* both the female and the male function can gain fitness by either outcrossing and selfing, outcrossing is presumably the preferred route to fitness over selfing for both functions, due to the previously mentioned delayed selfing and the potential for inbreeding depression (Ramm et al. 2012).

To investigate the initiation of selfing in *M. hystrix*, we wanted to explore whether a focal worm will initiate selfing if it is allowed to outcross via its male function (i.e. it can donate sperm to a partner), but at the same time denied the ability to outcross via its female function (i.e. it cannot receive sperm of a partner). Or in other words, we created a social environment, where the focal worm had access to only one type of gamete to outcross, namely the partner’s female gametes, while the access to the partner’s male gametes was experimentally prevented. Any fitness gain via the female function of such a focal worm was therefore restricted to selfing. In order to achieve this condition, we paired focal worms with experimentally manipulated partners, which lacked the stylet and were therefore unable to donate sperm to the focal worms (thus in some sense these experimentally manipulated partners resembled females). We achieved this by amputating the partner’s tail plate (including the male copulatory organ) to generate experimentally ‘emasculated’ worms, a treatment from which these flatworms readily recover within a few days, as we outline in some detail below.

Since a very rapid regeneration of the stylet in experimentally emasculated *M. hystrix* might potentially compromise our experimental aim of preventing our focal worms from gaining access to allosperm, we first performed a preliminary experiment in which we investigated the regenerative capabilities of *M. hystrix*. In particular, we described the required regeneration time of the tail plate in *M. hystrix*, including the formation of the male copulatory organ and its connection to the seminal vesicle, the reproductive structure containing own sperm ready to be donated.

By comparing experimental focal worms paired with emasculated partners to control focal worms paired with either unmanipulated control partners and control focal worms held in isolation, we could test, on the one hand, the hypothesis that experimental focal worms will initiate selfing, since the initiation of selfing is primarily linked to the female function, due to a lack of received sperm. On the other hand, however, if a lack in sperm donation associated with the male function is the important factor, we expected that the experimental focal worm will not initiate selfing.

## Methods

### Study organism

As the study organism, we used the free-living flatworm *Macrostomum hystrix* (currently best referred to as *Macrostomum hystrix* Ørsted 1843 sensu Luther 1905; see Schärer et al. 2011 for further taxonomic information). In the laboratory, *M. hystrix* is kept in mass cultures of 100 animals per glass Petri dish containing 20 ml of artificial sea water (ASW) at a salinity of 6‰ (w/w) and fed ad libitum with the diatom algae *Nitzschia curvilineata*. Under these conditions, the generation time is approximately 25 days (Ramm et al. 2012). The culture maintained in the laboratory is an inbred line, called SR1 (Zadesenets et al. 2016; Winkler and Ramm 2018), which was started in 2011 based on an outbred *M. hystrix* culture, which initially derived from worms collected in May 2010 from the San Rossore Regional Park (43.684° N, 10.283° E) near Pisa, Italy. Little is currently known about the natural ecology of *M. hystrix*, and no studies have been done on natural selfing rates to date. However, given that this species has evolved an unusual and intriguing route to selfing, namely hypodermic self-insemination after a considerable waiting time, it seems likely that selfing is a common feature of its life history.

### Timing of tail plate regeneration

Flatworms such as *Macrostomum lignano*, a close relative to *M. hystrix* (Schärer et al. 2011)—as well as the more distantly related planarian flatworms (Wagner et al. 2011)—are known for their impressive regenerative abilities (Ladurner et al. 2000; Nimeth et al. 2006; Egger et al. 2007; Lengerer et al. 2016). For example, *M. lignano* is capable of regenerating its tail plate within 6 to 10 days after amputation (Egger et al. 2006; Egger et al. 2009), thanks to a powerful stem cell system that consists of so-called neoblasts (Wagner et al. 2011; Ladurner et al. 2008). In *Macrostomum*, the regeneration process involves the formation of a cell mass capable of growth (a blastema), in which many neoblasts proliferate extensively and later differentiate into a range of different cell types (Egger et al. 2009), finally forming the different organ systems shown in Fig. 1.

In order to determine the minimum time required for *M. hystrix* to regenerate its tail plate, we performed a preliminary experiment in which we amputated the tail plates of 60 worms and followed their regeneration process over 5 days (details on the methods, results and discussion of this preliminary experiment are given in the Online Resource: tail regeneration). Briefly, after 4 days of regeneration (Online Resource: Fig. ESM1), we observed the first mature stylets connected to the seminal vesicle (Online Resource: Fig. ESM2), which suggests for our ‘Initiation of selfing’ experiment that a focal *M. hystrix* should be paired with an experimentally emasculated partner for no longer than 3 days.

### Initiation of selfing

#### Experimental design

**Fig. 2:**
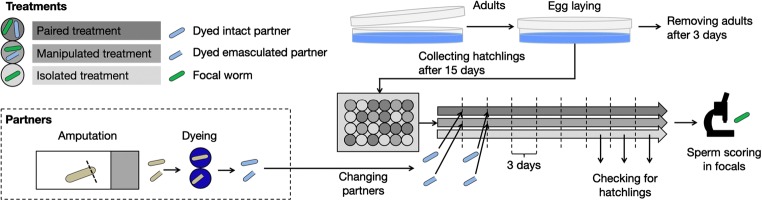
Overview over the experimental design to examine the initiation of selfing in *Macrostomum hystrix*. Paired focals and manipulated focals obtained new partners every 3 days, in order to ensure these retained their blue dye and the emasculated state. Isolated focals were regularly monitored for the presence of hatchlings as an estimate for the initiation of selfing. Once the majority of isolated focals had initiated selfing, all focals were scored for the presence of parenchymal sperm. Based on the spatial distribution pattern of sperm, we made an inference on whether the sperm had been injected by a partner or self-injected by the focal worm itself, and thereby distinguished between outcrossing and selfing. Initial sample sizes for all three treatments were *n* = 64 each

In order to explore the reproductive strategy of *M. hystrix* in different social environments, we assigned focal worms to one of three different treatments (isolated, manipulated and paired). In the isolated treatment, we kept the focal worms in isolation, such that these ‘isolated focals’ had no access to gametes of a partner and were therefore expected to self-fertilize by injecting their own sperm into their anterior body regions (Ramm et al. 2015). In the paired treatment, we obtained ‘paired focals’ by pairing worms with intact partners (dyed blue to allow distinction, see below for details). In this treatment, outcrossing is expected, since a mating partner is available and outcrossing is likely the preferred reproductive strategy in *M. hystrix* (Ramm et al. 2012). Finally, in the manipulated treatment, focal worms were paired with experimentally emasculated partners (also dyed blue). Such focals are in the following referred to as ‘manipulated focals’, indicating that they were paired with manipulated partners, but note that they were not directly manipulated themselves. The experimental design is summarized in Fig. 2. Experimentally emasculated partners are still hermaphroditic in the sense that they still produce both male and female gametes, but they lack the male copulatory organ. So, in contrast to the intact partners of the paired treatment, the experimentally emasculated partners are unable to donate sperm to the thereby manipulated focals. Consequently, while manipulated focals can use their male gametes to outcross, the fertilization of their own female gametes depends on selfing.

We consider two possible scenarios for the behaviour of the manipulated focals. On the one hand, a manipulated focal might initiate selfing when its eggs are not being fertilized, since it does not receive sperm from emasculated partners. In this case, a manipulated focal would be expected to behave like an isolated focal. On the other hand, a manipulated focal might fail to detect that its eggs are not being fertilized and might therefore not initiate selfing, since its male function is given the opportunity to outcross via donating sperm to the experimentally emasculated partner. In this case a manipulated focal would be expected to behave in a way similar to a paired focal. While the first scenario would indicate that the initiation of selfing is linked to the female function (i.e. selfing occurs due to a lack of sperm receipt by the focal), the second scenario would suggest a link between the male function and the initiation of selfing (i.e. selfing occurs due to a lack of opportunities for sperm donation by the focal).

#### Experimental protocols

To rear the partner worms, 360 adult *M. hystrix* from the mass cultures were pooled and randomly distributed into three Petri dishes containing ASW and algae. We kept the adults in these dishes for 3 days in order for them to lay eggs and produce a first batch of offspring to be used as the partner worms. Afterwards, all adult animals were transferred to three new dishes to produce a second partner batch. We followed this procedure, using the same parents, until we ended up with ten batches of partner worms (i.e. 10 times 3 dishes). As each partner batch reached an age of about 4 weeks, it was transferred into fresh dishes to ensure good growth conditions. We used the same parental worms to produce the focal worms, and once again, we removed the adults after 3 days of egg laying. Fifteen days later, we pooled the still immature focal worms and randomly distributed 192 focals into eight 24-well plates, one focal in each well. The 24-well plates were prepared beforehand, containing 1.5 ml ASW with ad libitum algae. At this stage (day 0 of our experiment), all focals had reached an age of 16.5 (± 1.5) days. We assigned each well randomly to one of the three treatments in a balanced way.

Over the course of the following experiment, the manipulated and paired focals were kept together with a partner. On day 1 of the experiment, we pooled all partner worms of the first batch, which by this time had reached maturity and an age of 48.5 (± 1.5) days (note the difference in age between focal and partner worms, which assured that focals were always paired with a mature worm, even if they had not yet matured themselves). Alternatingly, a mature partner for the paired and manipulated treatment, respectively, was prepared, until we ended up with about 70 partners for each treatment. For the manipulated treatment, we transferred each partner onto a microscope slide and amputated the tail plate, including the stylet, using a surgical knife (see Online Resources: ‘Tail plate regeneration’). By amputating the stylet, we obtained partner worms in a temporarily emasculated state. Partner worms of the paired treatment were treated identically, except for the fact that they were not amputated.

In order to distinguish between the partners and the focals later in the experiment, we dyed the partners by placing them for 6 h into 10 μl of a solution of a food dye (E131, Trawosa AG; 3 mg/ml ASW; filtered through a paper filter to remove undissolved particles) in the well of a Terasaki loading plate (Greiner Bio-One International, 653102). The same food dye has previously been used on *M. lignano* without showing any negative effects on the dyed worms (Marie-Orleach et al. 2013). Moreover, the higher concentration of the dye used in this study, which was necessary to dye worms for an entire period of 3 days, was tested in a preliminary experiment on both regular and amputated *M. hystrix* without signs of adverse effects (data not shown). Once all partners were dyed, we distributed them to the focals of the respective treatment in the 24-well plates.

On day 4 of the experiment, the partners of the second batch were prepared in the same way as those of the first (note that the timing of the preparation of the replacement batches was informed by the results of the ‘Tail plate regeneration’ experiment). While dyeing the partners of the replacement batch, we removed the first batch partners from the 24-well plates. We transferred both focal and partner onto a microscope slide, which allowed us to identify and remove the partner based on its blue colour. We then transferred the focals back to their wells and added the dyed partners of the replacement batch. To keep the conditions for all focals similar, we also transferred the isolated focals onto microscope slides and back. Additionally, we checked the wells of the isolated focals for progeny to check for the initiation of selfing. We continued the abovementioned procedure of exchanging partners and checking for progeny in a 3-day interval until the majority of isolated focals had started to produce offspring, namely on day 19 of the experiment. By this time the seventh batch of partners was added. Moreover, every 9 days we transferred all worms into fresh 24-well plates.

#### Sperm scoring

As a proxy for the reproductive strategy (i.e. selfing vs. outcrossing) of focals in the three treatments, we scored the spatial distribution of injected parenchymal sperm cells. Due to the large sample sizes, sperm scoring was split over 2 days, which were balanced for all treatments. Specifically, sperm scoring was done on days 20 and 21 of the experiment (i.e. age of the focal worms 35 to 38 and 36 to 39 days, respectively). We isolated all focals and transferred them into a new 24-well plate in order to blind the observer with regard to the three treatments. Focal worms were anesthetized in a 1:4 mixture of MgCl_2_ (7.14% in H_2_O) and ASW for approximately 8 min before dorsoventrally squeezing them in 40 μl ASW between a glass slide and a coverslip (21 × 26 mm coverslip), using small plasticine feet as spacers, to increase the visibility of anatomical structures and to prevent excessive movement of the worms during examination. Worms were scored using differential interference contrast microscopy at × 100, × 400 and × 1000 magnification. We scored parenchymal sperm in three different body regions of the squeezed worm, namely the head, gonad and tail region (Fig. 1), similar to the sperm scoring described in Ramm et al. (2015). The amount of parenchymal sperm was scored in each body region for approximately 1 min, according to the following ordinal scale (0 = no sperm, 1 = one up to ten sperms, 2 = eleven up to thirty sperms, 3 = more than thirty sperms). In parallel to sperm scoring, we also scored the number of developing eggs in the focals (which did not differ significantly between the treatments, see Online Resource Fig. ESM3) and checked for the presence of the stylet and gonads to ascertain whether all scored focals had reached maturity (which they all had).

#### Statistics

Two replicates were dropped from the analyses since the number of adult worms found in a well did not match the expected number for the respective treatment. Moreover, in one paired treatment, the blue dye of the partner was not recognizable after 3 days, so this replicate was also excluded from the experiment. And finally, a few replicates were lost during handling. The final sample size was *n* = 59 for the isolated, *n* = 57 for the manipulated, and *n* = 55 for the paired treatment.

In order to compare the sperm distribution patterns between the treatments, we needed an estimate that simultaneously accounts for the possible statistical dependence of the three sperm scores obtained from the head, gonad and tail region in each specimen and also the ordinal nature of the sperm scores. We used the same non-parametric test as Ramm et al. (2015) in their study on sperm distribution patterns in *M. hystrix*. Briefly, the Spearman’s rank correlation coefficient (*r*_*s*_) allows assessing the correlation between two non-continuous variables, which in our case was the correlation between the ranks of the observed distribution and the ranks of the expected sperm distribution for outcrossing *M. hystrix* based on earlier findings (cf. Ramm et al. 2015). Specifically, Ramm et al. (2015) found that grouped *M. hystrix* had the highest sperm scores in the tail region (rank 1), intermediate sperm scores in the gonad region (rank 2) and the lowest sperm scores in the head region (rank 3). We thus expected positive Spearman’s rank correlation coefficients (*r*_*s*_ > 0) for paired focals (since they are expected to outcross, leading to high scores in the tail region), while we expected negative correlation coefficients (*r*_*s*_ < 0) for isolated focals (since they can only self-fertilize, leading to high scores in the anterior regions). Note that the Spearman’s rank correlation coefficients could not be calculated directly for 32 out of 171 specimens (19%) since the observed sperm scores did not show any variation across the three body regions. In these cases we assigned a Spearman’s rank correlation coefficient of zero (*r*_*s*_ = 0) (cf. Ramm et al. 2015).

We then compared the *r*_*s*_ values of the three treatments using a Kruskal-Wallis test, followed by a post hoc Dunn’s multiple comparison test, correcting for false discovery rate via Benjamini-Hochberg adjustment. Similarly, we also compared an estimate of the total amount of observed parenchymal sperm, using the total sperm score of each specimen (i.e. the sum of the sperm scores across all three body regions). However, considerable care is needed to interpret these total sperm scores, as summing these ordinal-scale scores of course does not represent a linear measure of the amount of parenchymal sperm. All statistical analyses were done using the software R version 3.3.1. (R Core Team 2016).

## Results

**Fig. 3:**
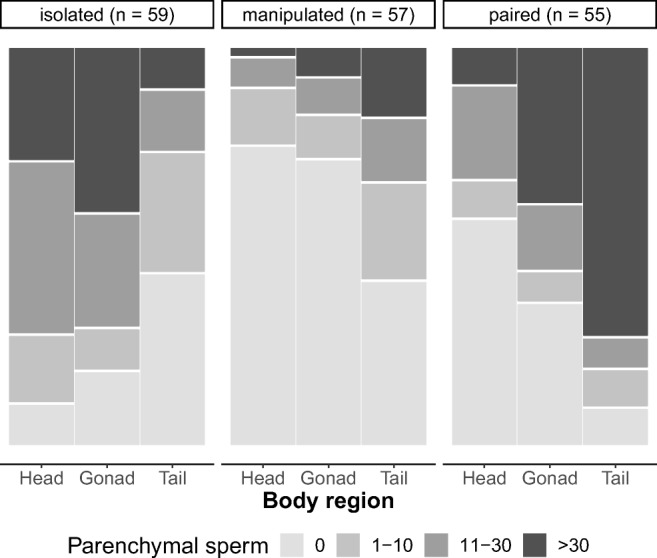
Sperm distribution patterns in *Macrostomum hystrix* reared in three different social environments. Note that different shades of grey represent the sperm score values (i.e. lightest grey = sperm score 0, darkest grey = sperm score 3)

After having been raised in different social environments, we observed distinct sperm distribution patterns in focal worms from each treatment (Fig. 3). In isolated focals the sperm scores tended to decrease from the head to the tail region, while in paired focals, the values tended to increase towards the tail region, closely mirroring earlier results for these two treatments (Ramm et al. 2015). In contrast, the manipulated focals showed relatively low values across all body regions.

**Fig. 4:**
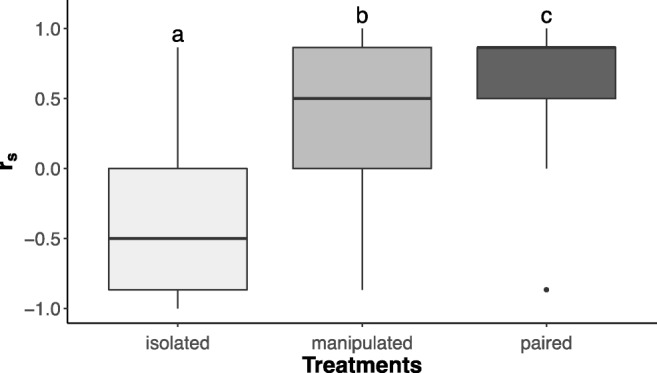
Spearman’s rank correlation coefficients for the sperm scores in the three treatment. Box plots indicate the 25th percentiles, medians and the 75th percentiles, and the whiskers extend the boxes by 1.5 times the interquartile range. Different letters denote statistically significant differences between the treatments after post hoc testing (see ‘Results’)

The Spearman’s rank correlation coefficients for the associations between ranks of the observed sperm scores of each specimen and the ranks of the expected sperm scores for outcrossing *M. hystrix* are shown for each treatment group in Fig. 4. We found a positive median *r*_*s*_ in paired focals, while isolated focals showed a negative median *r*_*s*_, thus similarly confirming earlier results for these two treatments (Ramm et al. 2015). In the manipulated focals, we also found a positive, albeit lower, median *r*_*s*_, thus more closely resembling the pattern observed in paired focals. Overall, the Kruskal-Wallis test revealed a significant difference for *r*_*s*_ between the treatments (DF = 2, *χ*^2^ = 63.57, *p* value < 0.001). Moreover, Dunn’s multiple comparison post-hoc tests revealed that all treatments were significantly different from each other (Fig. 4; manipulated vs. isolated, *Z* = 5.73, *p* value < 0.001; manipulated vs. paired, *Z* = − 1.94, *p* value = 0.026; isolated vs. paired, *Z* = 7.63, *p* value < 0.001).

**Fig. 5:**
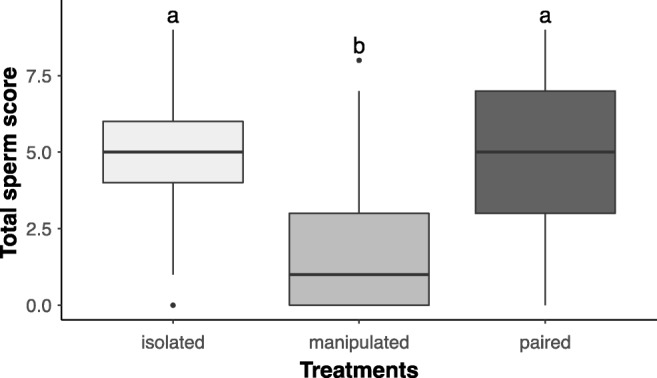
Total sperm score distributions across the three treatments. Total sperm score was calculated for each specimen by summing the sperm scores of each body region (head, gonads and tail). Note that care is needed to interpret these total sperm scores, as summing these ordinal-scale scores does not represent a linear measure of the amount of sperm. Box plots indicate the 25th percentiles, medians and the 75th percentiles, and the whiskers extend the boxes by 1.5 times the interquartile range. Different letters denote statistically significant differences between the treatments after post hoc testing (see ‘Results’)

And finally, we compared the total sperm scores (Fig. 5) across all treatments using the Kruskal-Wallis test, revealing a significant difference between the treatments (DF = 2, *χ*^2^ = 45.29, *p* value < 0.001). The Dunn’s multiple comparison post hoc test showed that the manipulated focals had significantly lower total sperm scores, and thus likely fewer parenchymal sperm than both isolated (*Z* = − 5.74, *p* value < 0.001) and paired focals (*Z* = − 5.91, *p* value < 0.001), while isolated and paired focals did not differ significantly in total sperm score (*Z* = 0.27, *p* value = 0.394).

## Discussion

Our study suggests that the majority of the manipulated focals probably did not initiate selfing over the course of our experiment, or if they did, that they did so at a much lower rate than the isolated worms (see the following section for a more detailed discussion). As a brief reminder, our hypotheses for the behaviour of the manipulated focals we paired with experimentally emasculated partners were either (i) that such focals initiate selfing to secure fertilization of their own eggs (selfing due to a lack of sperm receipt) or (ii) that such focals fail to realize the lack of received sperm and do not initiate selfing since they are able to engage in sperm donation. Therefore, our results suggest that selfing is more likely initiated by a lack of sperm donation, and therefore linked more to the male function, rather than being due to a lack of sperm receipt, and therefore less linked to the female function.

Knowing that the initiation of selfing may be (primarily) linked to one sex function might help to better understand the reproductive strategies of hermaphroditic individuals that find themselves in a situation where the fitness gains through the initiation of selfing for the male and female function are in opposition. For example, consider a scenario in which a focal individual has access to sperm from a partner (i.e. fitness gain via the female function through outcrossing) but never manages to donate sperm (i.e. no fitness gain via the male function through outcrossing). It appears possible that such a focal, linked to the male drive to donate sperm, would initiate selfing even if this lowered its overall fitness. More specifically, by initiating selfing, such a focal could increase its fitness via the male function, but at the same time, it might decrease its fitness via the female function, and thus possibly the overall fitness, by potentially introducing inbreeding depression, as injected autosperm compete with allosperm for fertilization of the own eggs (i.e. producing offspring of lesser quality).

In contrast to our findings, in plants, selfing sometimes appears to be linked to a lack of pollen receipt (Goodwillie and Weber 2018). For example, in the herbaceous plant, *Kosteletzkya virginica* (Malvaceae), stigmas that fail to receive pollen initiate selfing by curving their style towards their own pollen, while stigmas that have received pollen do not show this behaviour, likely via a signal resulting from pollen tube growth (Ruan et al. 2008). Compared to what we find in our flatworms, selfing linked to a lack of pollen receipt appears to create a different dynamic. Consider a similar scenario in which a focal flower has access to fitness gain via the male function through donating pollen, but never manages to receive pollen (i.e. no fitness gain via the female function through outcrossing). Under the assumption of negligible costs of pollen discounting, the initiation of selfing via the female function does not interfere with potential outcrossing opportunities through the male function and thus creating less potential for conflict between selfing versus outcrossing. In other words, selfing due to a lack of pollen receipt seems less likely to lower an individual’s overall fitness in such a scenario. In contrast to direct contact with a mating partner in *M. hystrix*, pollen donation in plants is often facilitated through pollinators or wind. Thus, pollen donation is not directly associated with the availability of a partner and thus the lack of pollen donation might be a poor signal to initiate selfing in plants. Additionally, Bateman’s principle applied to hermaphrodites, creates the expectation that hermaphrodites seek to mate in the male role more commonly than mating in the female role (Charnov 1979; Anthes et al. 2010; Schärer et al. 2014). Under this assumption, sperm limitation seems less likely in animals that engage actively in mating compared to plants, where pollen limitation has shown to be common (reviewed in Burd 1994). Instead, such animals may more often be in a situation where they tend to obtain too much sperm (Charnov 1979; Michiels 1998), and they may therefore not pay much attention to whether they have received any sperm.

We want to emphasize, however, that although our findings support the idea that the initiation of selfing is primarily linked to the male function, we lack data on how a focal worm would behave under reciprocal conditions to our experiment (i.e. conditions under which the focal worm has access to allosperm, can donate sperm to self-fertilize, but cannot donate sperm to a partner). Our approach of mechanically removing the stylet would not be suitable to obtain this reciprocal condition, since removing the stylet of the focal worm would not only prevent sperm donation to a partner, but would also prevent self-insemination. Currently, we do not see how this reciprocal condition could be achieved experimentally.

Moreover, another potential caveat for interpreting our results is the possibility that manipulated focals delayed selfing for a longer time period than the isolated worms to which they were compared (i.e. beyond the time period covered in our experiment). Immature focals were 17.5 (± 1.5) days old when they were first exposed to different social environments for either 19 or 20 days, reaching an average age of 37 (± 2) days at the end of our experiment. Given an estimated generation time of 25 days and an average waiting time for selfing in isolation of approximately 7 days (Ramm et al. 2012), we would have expected all focal worms to have had sufficient time to grow sexually mature as well as having ample opportunity to become reproductively active while exposed to their respective social environments. In order to monitor the initiation of selfing in the focals, we regularly searched the wells of the isolated focals for offspring. A few days before measuring, 39 out of 64 isolated focals had already started to produce progeny, giving us confidence to start scoring sperm 2 days later. This would mean that manipulated focals would have to delay selfing by a least several additional days compared to isolated worms.

For the isolated focals, we did find on average high sperm scores in the head and gonad regions, while the scores in the tail region were generally low (Fig. 3), and these findings did meet our expectations for selfing *M. hystrix* (Ramm et al. 2015). Except for two individuals, parenchymal sperm was observed in all isolated specimens (Online Resource Fig. ESM4 a-d), which, alongside the high percentage of offspring found in this treatment group, clearly suggested selfing activity of isolated focals. In contrast, paired focals did reveal a distribution pattern for parenchymal sperm with low scores in the head region, intermediate scores in the gonad region and the highest scores in the tail region (Fig. 3; Fig. ESM4 i-l), which also matched our expectations for outcrossing worms based on Ramm et al. (2015). In manipulated focals, we observed low sperm scores across all body regions (Fig. 3; Fig. ESM4 e-h). Overall, sperm scores in manipulated focals resembled neither the pattern of selfing worms (high scores in the head region) nor the pattern for outcrossing worms (high scores in the tail region). While low sperm scores in the tail region were expected due to the pairing of manipulated focals with experimentally emasculated partners, it shows that our experimental setup clearly did limit access to allosperm, as intended. However, in five manipulated focals we observed the highest sperm scores in the tail region and lowest in the head region (note that only five of the ten manipulated focals depicted in Fig. ESM4 h show this pattern). This specific sperm distribution pattern was not seen in any of the isolated focals without access to allosperm, but was present in many of the paired focals. Based on these findings, we need to consider the possibility that five manipulated focals did manage to receive sperm from their partners at some point throughout the course of the experiment. However, since this number (5/57) is quite low, we do not expect a substantial effect on our overall conclusions.

Selfing in *M. hystrix* has been suggested to be performed via hypodermic insemination into the anterior body regions (Ramm et al. 2015). Therefore, to find parenchymal sperm in the tail region of isolated and manipulated focals might seem surprising, given the intended exclusion of their ability to receive sperm from a partner. However, some parenchymal sperm in the tail region of isolated *M. hystrix* had already been found in a previous study (Ramm et al. 2015). We therefore want to briefly discuss two possible explanations for our findings of parenchymal sperm in the tail region of isolated and manipulated focals, other than outcrossing. Traumatically inseminated sperm has been shown to be able to migrate to the site of fertilization in terrestrial arthropods (Tatarnic et al. 2014). And also in *M. hystrix*, sperm is presumably capable of migrating through tissue towards the eggs, possibly facilitated by its less complex morphology and small size compared to sperm of *M. lignano* (Schärer et al. 2011). Since fertilization most likely happens near the boundary between the gonad and tail regions where the developing eggs are located (Fig. 1), injected sperm is expected to travel from the anterior body regions towards the tail region. Moreover, particularly in focals with low sperm scores in the tail region, we often observed sperm in the tissue near the male antrum, as well as in the male antrum itself (the male antrum is the canal in which the stylet resides when it is retracted). It appears possible that ejaculated sperm might occasionally swim back up in the male antrum, be released from the stylet while the stylet is still inside of the male antrum, and/or manage to penetrate the epidermis and enter surrounding tissues.

## Outlook

For further investigations, it would be interesting to focus on the interaction between the manipulated focals and their experimentally emasculated partners. We so far lack evidence whether or not manipulated focals actually transferred sperm to their experimentally emasculated partners and therefore gain fitness via outcrossing by the male function. In order to understand the reproductive choices of *M. hystrix* better, it would be desirable to be able to observe and distinguish received sperm from different donors in vivo in experimentally emasculated partners (possibly by generating transgenic worms as in *M. lignano*, Marie-Orleach et al. 2016, 2017) or by using the size of seminal vesicles of the focals under standardized squeezing as an estimate for their patterns sperm donation and production (e.g. Schärer and Ladurner 2003; Schärer and Vizoso 2007). Moreover, further data about what triggers the initiation of selfing in other species is central in order to learn whether the suggested link to the lack of sperm donation is consistent across species.

## Electronic supplementary material

ESM 1 (DOCX 6954 kb)

## Data Availability

Data is provided as supplementary material.
