# Supplementary material for: Is the initiation of selfing linked to a hermaphrodite’s female or male reproductive function?
Source: Behav Ecol Sociobiol. 2020 Mar 17;74(4):41. doi: 10.1007/s00265-020-2816-3 (PMC7080307; doi:10.1007/s00265-020-2816-3)
Supplement: Supplementary file 1 — (DOCX 6954 kb) [file 265_2020_2816_MOESM1_ESM.docx]

# Electronic Supplementary Material

Article tile:

Is the initiation of selfing linked to a hermaphrodite’s female or male reproductive function?

Journal name:

Behavioral Ecology and Sociobiology

Author names:

Philipp Kaufmann & Lukas Schärer

Affiliation and e-mail of the corresponding author:

Evolutionary Biology, Zoological Institute, University of Basel, Vesalgasse 1, CH-4051 Basel, Switzerland

philipp.kaufmann@ebc.uu.se

**Timing of tail plate regeneration**

***Methods***

Experimental setup

In order to determine the minimum time required for *M. hystrix* to regenerate its tail plate, we amputated the tail plates of 60 worms and followed their regeneration process over five days. We picked five- to six-week-old worms from the mass culture and verified their maturity, checking for the presence of gonads, a stylet and a complete tail plate (normal shape and presence of adhesive organs). All worms were investigated using a Leica DM 2500 light microscope (using differential interference contrast at both 100x and 400x magnification). The worms were prepared by dorsoventrally squeezing them in 40 µl ASW between a glass slide and a coverslip (21 x 26 mm coverslip), using small plasticine feet as spacers, to increase the visibility of anatomical structures and to prevent excessive movement of the worms during examination. We then isolated the worms in wells of 24-well plates (TPP, Switzerland) containing ASW and *ad libitum* algae.

After two days of isolation, we transferred the worms in 4 µl of ASW onto a glass slide and made a cut at the level posterior to the female antrum (the female genital organ responsible for egg laying; fig. 1) using a surgical knife, cutting through the seminal vesicle and thus amputating their tail plate, including the stylet. After cutting, the amputated worms were put back into their well plate, and the amputated tail plate was investigated via light microscopy in order to check for successful stylet removal.

The amputated worms were then randomly (2/3 vs. 1/3) split into a daily-monitored treatment (n = 42 worms; measured on days 1, 2, 3, 4, and 5 after amputation) and a control treatment (n = 18 worms; measured only on day 5 after amputation). This setup allowed us to detect potential influences of the daily handling—which could potentially harm the worms—on the timing and success of the regeneration process.

Scoring of regeneration

To identify the time required for the regeneration of the tail plate in *M. hystrix*, we followed the regeneration of the stylet and its connection to the seminal vesicle as estimates for regained sexual maturity after the amputation. The scoring of the regeneration process was done by investigating squeeze preparations (as outlined above), documenting the following parameters: presence of the stylet (0 = no stylet, 1 = forming stylet, 2 = mature stylet) and the connection of the seminal vesicle to the regenerated stylet (0 = not connected, 1 = connected). Moreover, we also considered a combined score, where a specimen with a regenerated mature stylet connected to the seminal vesicle was considered fully recovered (1), while other worms were considered not yet fully recovered (0). Note that scoring on day 5 was done blind with respect to the treatment.

Statistics

Some replicates had to be excluded from analysis, because the cutting level was off target or because there was uncertainty about the effectiveness of the removal of the stylet (e.g. when the stylet was not found in the amputated tail plate). Additionally, some replicates were lost during handling over the course of the experiment, resulting in a final sample size of n = 33 for the daily-monitored treatment and n = 15 for the control treatment.

To test whether daily-monitoring has an impact on the regeneration time, we used Fisher’s exact tests, comparing the stylet regeneration and its connection to the seminal vesicle between the two treatments after five days of regeneration. Specifically, we compared the frequencies of specimens showing i) either no stylet, a forming stylet or a mature stylet using a 2x3 Fisher’s exact test, ii) either no connection or a connection of the seminal vesicle with the regenerated stylet, and iii) either not yet recovered or fully recovered worms between the two treatments, both using 2x2 Fisher’s exact tests. All statistical analyses were done using the R version 3.3.1.

## ***Results***

The general process of regeneration observed over the course of the experiment is shown in the supplementary information (Online Resource fig. ESM1). We observed the first mature stylets after four days of regeneration, while one day earlier a similar percentage of investigated specimens showed forming stylets (Online Resource fig. ESM2 a). Moreover, we compared the scored stylet regeneration values on day 5 between the daily-monitored treatment and the control treatment, revealing no significant difference (two-sided 2x3 Fisher’s exact test: *p*-value = 0.442). While the majority of regenerated mature stylet were connected to the seminal vesicle (Online Resource fig. ESM2 b), we observed the connection of the seminal vesicle to a forming stylet only once, in a specimen of the control treatment. There was no significant difference in the proportion of specimens with a seminal vesicle connected to a regenerated stylet (forming stylet or mature stylet) on day 5 between both treatments (two sided 2x2 Fisher’s exact test: *p*-value = 0.757). And finally, over the course of five days 13 out of the 33 specimens of the daily-monitored treatment recovered fully (i.e. having a mature stylet connected to the seminal vesicle), while in the control treatment 4 out of 15 specimens recovered fully (two-sided 2x2 Fisher’s exact test: *p*-value = 0.521).

These findings suggest that daily-monitoring does not delay regeneration and we concluded for our “Initiation of selfing” experiment that a focal should be paired with an experimentally ‘emasculated’ partner for no longer than three days.

## ***Discussion***

Although not specifically tested in this study, we presume that the regeneration of the tail plate of a previously mature *M. hystrix* will result in a mature individual that is able to transfer sperm and therefore will be fully fertile. This assumption is based on the findings of Egger et al. (2006) in *M. lignano*, where they could show that specimens were able to sire offspring once they fully recovered from having their gonads and stylet amputated. We showed that *M. hystrix* is capable of regenerating a mature stylet within four days after amputation and observed tail plates with forming stylets one day earlier. Our results, together with the assumption of fertility of recovered worms, leads to the conclusion that a previously amputated *M. hystrix* is potentially capable of donating sperm within four days of regeneration. This is the central result of this preliminary experiment with regards to the design of the “Initiation of selfing” experiment, where we therefore exchanged the partners every three days. Note that we derived the minimum required recovery time of the stylet from the daily-monitored treatment, which was handled daily and therefore could potentially have delayed the regeneration process. However, since there were no significant differences between the daily-monitored treatment and the control group on day 5, we assume that the minimum recovery time of four days is applicable also to the control group and hence also for experimentally ‘emasculated’ partners in the “Initiation of selfing” experiment (which were also handled less frequently than the worms of the daily-monitored treatment).

We observed the first regenerated stylets (forming stylets or mature stylets) to be connected to the seminal vesicle also on day four, supporting our assumption that previously amputated *M. hystrix* may potentially transfer sperm from day four onwards. In most specimens that showed a regenerated mature stylet after five days of regeneration, the stylet was also found to be connected to the seminal vesicle, regardless of the treatments (daily-monitored treatment, 13 of 16, 81.3%; control treatment, 4 of 6, 66.7%). This tells us that the majority of regenerated mature stylets in *M. hystrix* are connected to the seminal vesicle and further suggests sexual maturity of the regenerated specimens. In all but one of the cases, the connection was exclusively found in specimens with a mature stylet. Note that the connection between the seminal vesicle and a regenerated stylet is probably easier to detect in specimen with a mature stylet and a seminal vesicle full with sperm than in specimen with a forming stylet and a seminal vesicle with less sperm. During the experimental time frame of five days, roughly 45% of the flatworms regenerated to sexual maturity, suggesting that there is considerable temporal variation in the regeneration process. Our focus here was on the minimal time required for regeneration and thus only on the early spectrum of this variation. In future experiments it would be interesting to explore what influences this variation in regeneration time, and using multiple amputations one could study whether the speed of regeneration is an individual trait.


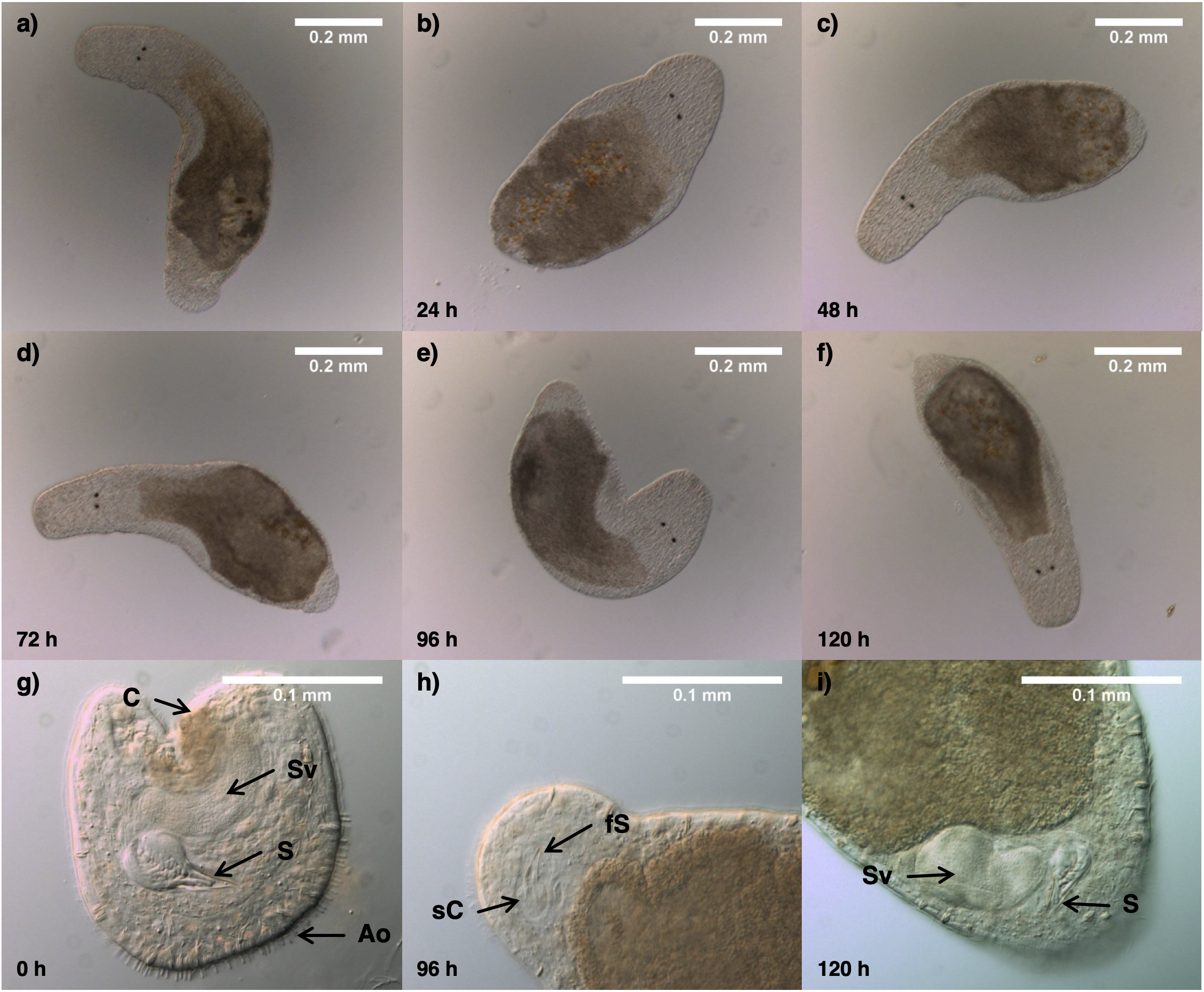


Fig. ESM1 Regeneration process of an individual daily-monitored *Macrostomum hystrix* over the course of five days after amputating its tail plate. In the bottom left corner is shown the time since the amputation. a) Specimen two days before amputation, confirming its maturity and serving as a reference for comparison during the regeneration process. b) Successfully closed wound. c) Formation of small blastema. d) Larger blastema. e) Partially regenerated tail plate (see also h). f) Fully regenerated tail plate (see also i). g) Freshly amputated tail plate displaying the cutting level (C), seminal vesicle (Sv), stylet (S) and adhesive organs (Ao). h) Regenerating tail plate after four days, showing a forming stylet (fS). Note the rounded structure at the base of the forming stylet consisting of stylet secreting cells (sC). i) Regenerated tail plate of a different specimen showing a fully regenerated stylet (S) connected to the seminal vesicle (Sv), resembling the tail plate before the amputation. The focal worm was considered fully recovered at this point

(Photographs, edited by PowerPoint)Fig. ESM2 Regeneration progress of anatomical structures in *Macrostomum hystrix* after amputation of the tail plate. a) Regeneration of the stylet. b) Connection of the seminal vesicle to the regenerated stylet. Four days after the manipulation, the first regenerated mature stylets were observed in the daily-monitored treatment, some of them already with a visible connection to the seminal vesicle

(R version 3.3.1 (R Core Team 2016))

Fig. ESM3 Number of developing eggs at the time of the sperm scoring. Note that the number of developing eggs was measured at a single time point during the sperm scoring (i.e. once at the end of the experiment) and does not capture the total amount of eggs produced during the course of the experiment. Box plots indicate the 25^th^ percentiles, medians and the 75^th^ percentiles, and the whiskers extend the boxes by 1.5 times the interquartile range. There was no significant difference between the three treatments (Kruskal-Wallis, DF = 2, ${}^{\boldsymbol{2}}$ = 3.30, p-value = 0.192)

**(**R version 3.3.1 (R Core Team 2016))

Fig. ESM4 Parenchymal sperm distributions in individual *Macrostomum hystrix* specimen categorized by treatment and tail region sperm scores. Sperm scores of the three body regions are connected by a line, allowing identification of typical patterns for selfing and outcrossing individuals. To avoid overly crowded graphs, the data is split not only by treatment, but also by the observed sperm score in the tail region. Additionally, to visualize the number of replicates with overlapping values, the points that the lines connect are slightly scattered in both the x- and y-direction. In the isolated focals (red, a-d), we observed many specimens that follow the overall pattern (fig. 4), namely with high scores in the head and gonad region and low scores in the tail region (a & b). However, a few individuals showed either low or high sperm scores across all regions (c & d). For the manipulated focals (blue, e-h) we found that the majority of specimens had low sperm scores across all regions (e & f), which again is not surprising given the overall pattern in fig. 4. However, this way of displaying the data reveals that a few individuals resembled patterns of presumably selfing isolated focals (e) and a few exhibited high sperm scores in the tail region with low scores in the anterior body regions (h), resembling predominantly outcrossing paired focals. And while we observed a few paired focals (green, i-l) with low sperm scores across all body regions (i & j), most showed high scores in the gonad and especially in the tail region (k & l). Finally, one paired focal showed a high sperm score in the head region, while scores for the gonad and tail regions were low (i), a pattern similar to isolated focals. This specimen is the only paired focal that showed a negative *r_s_* in fig. 5

(R version 3.3.1 (R Core Team 2016))
